# Commentary: the ideal patch material for congenital cardiac surgery: one ring to rule them all?

**DOI:** 10.1093/icvts/ivad027

**Published:** 2023-02-08

**Authors:** Supreet P Marathe

**Affiliations:** Queensland Paediatric Cardiac Service (QPCS), Queensland Children’s Hospital, Brisbane, QLD, Australia; School of Clinical Medicine, Children’s Health Queensland Clinical Unit, University of Queensland, Brisbane, QLD, Australia

**Keywords:** Patch material, Congenital heart surgery, Pulmonary artery augmentation

The repair of various congenital heart defects involves liberal use of patch material—from closing septal defects to augmenting the arch and pulmonary arteries. The ideal patch material should be readily available, have no specific storage requirements, be durable, non-thrombogenic, should be resistant to infection, calcification and aneurysm formation and should grow with the patient. A variety of patch materials have been tried and tested in congenital cardiac surgery including treated and untreated autologous pericardium, bovine pericardium, small intestinal submucosa, expanded tetrafluoroethylene and Dacron, to name a few [1]. Interest in these materials has waxed and waned over time. Needless to say, this is because none of the materials fulfil the ‘ideal’ requirements and the search for the best (if not ideal) patch material continues.

Adding to the list of such materials is decellularized equine pericardium commercially marketed as Matrix Patch™ (Auto Tissue Berlin GmbH, Berlin, Germany). Like several other patch materials in the past [2], the manufacturers describe a ‘favourable’ processing technique, which minimizes calcification and optimizes performance. Weixler *et al.* [3] from Berlin present their experience with the implantation of 341 equine pericardial patches in 201 patients with a median age of 2.5 years and a median follow-up of just under 2 years. Of note, >60% of the patches were used for reconstruction of either the right ventricular outflow tract or the pulmonary arteries (PA) and other sites formed the remaining 40% of patch implantation sites. In patients who had a follow-up >1 year, >25% of the patches needed reintervention and a vast majority of these (87%) were after augmentation of pulmonary arteries. Use for pulmonary artery augmentation was the only independent risk factor for reintervention. Considering the reputation of the unit, it is unlikely that surgical technical problems have played a role. The authors have also tried to evaluate handling characteristics by surgeons, thus introducing some subjectivity and equine pericardium seems to be excellent from that standpoint.

The findings by Weixler *et al.* are worrisome as pulmonary arteries are one of the commonest sites needing patch augmentation. The patch material usually does not make much difference when closing atrial or ventricular septal defects and boils down to individual preference. This study probably does not have enough patch numbers at other locations like valve repair and aorta/arch augmentation to have enough statistical power to tease out differences. As a surgeon, if a patch material is not working for pulmonary arteries which is a low-pressure circulation, I will be hesitant to use it for other locations, which will be more challenging to reintervene like the aortic arch or valve repair [4]. Having said that and as the authors stress, it is still early days in the evaluation of equine pericardium, and we hope to have more data coming from Berlin in the future to shed light on the long-term durability.

No patch material (including autologous pericardium) is perfect [5]. It is possible that there is no ‘one ring to rule them all’ and the ‘ideal’ patch material for different surgical sites is different. Studies like these at least tell us which material does not work in certain sites. Till large multicentre studies give us the answer or till we get a superior solution from tissue engineering, the pursuit of the ideal patch material will continue.
